# Pure Food

**Published:** 1880-09

**Authors:** 


### HALL'S JOURNAL OF HEALTH.

OUR LEGITIMATE SCOPE IS ALMOST BOUNDLESS: FOR WHATEVER BEGETS PLEASURABLE AND HARMLESS FEELINGS, PROMOTES HEALTH; AND WHATEVER INDUCES DISAGREEABLE SENSATIONS, ENGENDERS DISEASE.

### PURE FOOD.

It is no economy to use inferior food. It is a saving of money, and time and health, to give a higher price for what we eat, if it be fresh and perfect, than to obtain it for less on account of its being wilted, or old, or partially decayed.

Some people prefer to make their meat tender by keeping, which means that decomposition is taking place; in plainer phrase, it is rotting. Such meats require less chewing, and may appear very tender, but it is a physiological fact, that they are not digested as easily or as quickly as solid fresh meat.

When a vegetable begins to wilt, it is no longer that vegetable, because a change of particles has taken place, and in such proportion it is unnatural—it is dead—and to eat it tends to death.

One of the most horrible forms of disease is caused by eating sausages which have been kept a long time; more common in Germany than elsewhere. Scarcely anything saddens us so much in passing through some of the bye streets and the more eastern avenues, as the sight of the long kept meats and shrivelled vegetables, which are sold to the unfortunate poor at the corner Dutch groceries.

But the poverty stricken are not the only sufferers, the richest men come in for their share, for themselves and for their families in proportion, as the mistresses of their splendid mansions are incompetent or inattentive to those household duties, the proper performance or neglect of which makes all the difference between a true wife and a contemptible doll.

With all the high sounding advantages of high sounding "Young Ladies' Boarding Schools," and "Institutes" and all that, with all the twaddle about learning French and German, and music and æsthetics, how many of these paint-like girls are any more fit to take charge of a man's household than to navigate a ship, or calculate a parallax.

